# Prescribing medications of questionable benefit prior to death: a retrospective study on older nursing home residents with and without dementia in Germany

**DOI:** 10.1007/s00228-020-02859-3

**Published:** 2020-03-26

**Authors:** Christian Rausch, Falk Hoffmann

**Affiliations:** 1 Department of Health Services Research, Carl von Ossietzky University Oldenburg, Ammerländer Heerstraße 140, 26129 Oldenburg, Germany; 2 Department of Health Sciences, Community and Occupational Medicine, University of Groningen, University Medical Center Groningen, Antonius Deusinglaan 1, FA10, 9713 AV Groningen, The Netherlands; 3 Department of Global Public Health, Karolinska Institutet, Widerströmska huset, Tomtebodavägen 18A, SE, 17177 Stockholm, Sweden

**Keywords:** Dementia, Institutionalization, Inappropriate medications, Palliative care, Polypharmacy

## Abstract

**Purpose:**

We studied the prevalence of medications of questionable benefit in the last 6 months of life among older nursing home residents with and without dementia in Germany.

**Methods:**

A retrospective cohort study was conducted on claims data from 67,328 deceased nursing home residents aged 65+ years who were admitted between 2010 and 2014. We analyzed prescription regimens of medications of questionable benefit in the 180–91-day period and the 90-day period prior to death for residents with dementia (*n* = 29,052) and without dementia (*n* = 38,276). Factors associated with new prescriptions of medications of questionable benefit prior to death were analyzed using logistic regression models among all nursing home residents and stratified by dementia.

**Results:**

A higher proportion of nursing home residents with dementia were prescribed at least one medication of questionable benefit in the 180–91-day (29.6%) and 90-day (26.8%) periods prior to death, compared with residents without dementia (180–91 days, 22.8%; 90 days, 20.1%). Lipid-lowering agents were the most commonly prescribed medications. New prescriptions of medications of questionable benefit were more common among residents with dementia (9.8% vs. 8.7%). When excluding anti-dementia medication, new prescriptions of these medications were more common among residents without dementia (6.4% vs. 8.0%). The presence of dementia (odds ratio [OR] 1.40, 95% confidence interval [95%CI] 1.32–1.48) and excessive polypharmacy were associated with new prescriptions of medications of questionable benefit prior to death (OR 4.74, 95%CI 4.15–5.42).

**Conclusion:**

Even when accounting for anti-dementia prescriptions, the prevalence of nursing home residents with dementia receiving medications of questionable benefit is considerable and may require further attention.

**Electronic supplementary material:**

The online version of this article (10.1007/s00228-020-02859-3) contains supplementary material, which is available to authorized users.

## Introduction

The estimated prevalence of dementia among people aged 60 years and older is between 5 and 7% [1]. In 2010, around 5.6 million people lived with dementia worldwide, with numbers expected to rise to 65.7 million in 2030 [1]. Advanced dementia is characterized by severe cognitive impairment, functional decline, and multiple complications like pneumonia and dysphagia [2]. Thus, the progression of dementia often requires care in a nursing home, where individuals with advanced dementia often receive medical and palliative care at the end of life [2–6].

Medications are an important cornerstone in the management of dementia. However, a fine balance between harm and benefit exists, particularly when shifting from disease management to palliative care, with a focus on improved quality of life [7]. A majority of patients with dementia are older people, who are often burdened by more than one chronic condition [8, 9]. As such, individuals with dementia are often exposed to polypharmacy, i.e., a high number of medications. The risk of drug–drug and drug–disease interactions warrants cautious prescribing practices and guidelines to decrease the risk of adverse outcomes [10–12]. When shifting toward palliative care among older people with dementia, the primary focus becomes the prevention and management of suffering, comfort, as well as support of caregivers [2, 4]. With regard to the lack of guidelines in palliative care interventions in nursing homes and the risk of medication-related adverse events, a consensus panel has defined a list of medications considered to be “never appropriate” for older people with advanced dementia [13].

Various studies have shown that the prescription of medications of questionable benefit decreases in the terminal phase of nursing home residents with dementia [12, 14–17]. However, these studies also suggest that nursing home residents with dementia may still receive new prescriptions, including medications of questionable benefit, in the last months prior to death. Studies have considered the prevalence of these medications in older people with dementia in palliative care settings in Sweden, the USA, and other countries [12, 14, 15, 17]. Little is known about the situation in Germany and how prescription patterns of medications of questionable benefit compare between nursing home residents with and without dementia.

Therefore, this study aims to identify the prevalence of medications of questionable benefit in the last 6 months prior to death among older nursing home residents with and without dementia in Germany, in consideration of new prescriptions and associated factors.

## Methods

### Study design and sample

This retrospective cohort study of older people admitted to nursing homes between January 1, 2010, and December 31, 2014, has already been described in previous studies [18, 19]. Claims data of a German statutory health insurance (DAK-Gesundheit), which has an insured population of 6 million individuals, were used. The German Long-Term Care Insurance (*Gesetzliche Pflegeversicherung*) provided data on nursing home care, including information on level of care dependency, ranging from care level 1 (in need of care) to care level 3 (severe need of care).

### Study population

The study population included older people who were insured for at least 365 days without prior nursing home admission (*n* = 67,328). All people aged 65 years and older who, after nursing home admission, died on or before December 31, 2014, were considered in the final study population [19].

The study population included people with and without dementia. The presence of dementia was assessed using the ambulatory diagnosis of at least one relevant ICD code in the quarter of nursing home admission. In line with previous studies [20, 21], the following ICD-10 codes were used: F00.x, F01.x, F02.0, F02.3, F03, G30.x, G31.0, G31.1, G31.82, G31.9, and R54.

### Medications of questionable benefit

Information on medication prescribed was assessed through the five-level Anatomical Therapeutic and Chemical (ATC) classification system [22]. A previous study reported some of the medications prescribed in our study on a list of medications deemed “never appropriate” for older adults with advanced dementia [13]. Based on a modified Delphi process, a panel consisting of geriatricians decided on the medications to be included on the “never appropriate” list (see Supplementary Table 1.).

Newly prescribed and deprescribed medications of questionable benefit were defined based on the “never appropriate” medications defined by Holmes et al. [13], and prescriptions in the 180–91-day period were compared with those in the 90-day period prior to death. Newly prescribed was defined as any medication from one of the “never appropriate” groups having been prescribed within the 90-day period prior to death if no medication from these groups was prescribed in the 180- to 91-day period prior to death. Deprescription was defined in a similar way by the discontinuation of medications from any of the “never appropriate” groups in the 90-day period prior to death.

### Baseline characteristics

Age at death, sex, number of medications prescribed, length of stay in nursing home, and level of nursing care were considered baseline characteristics.

Age was categorized into the subgroups of 65–74 years, 75–84 years, 85–94 years, and 95 years and older.

The number of medications prescribed considered all medications on the ACT 5th level prescribed to an individual within 90 days of death. The number of medications prescribed was categorized into the previously described polypharmacy levels of 0–4 medications, 5–9 medications (minor polypharmacy), and 10 or more medications (excessive polypharmacy).

The length of stay in nursing home was counted from the first day of admission and grouped into quartiles.

The level of nursing care provides information on the level of dependency of those needing support in their activities of daily living, e.g., mobility, eating, and personal hygiene, as well as housekeeping. The three levels (1 to 3) correspond to the time required for assistance. A nursing care level of 1 represents some need of assistance, while level 3 represents the severest need of care [23].

### Statistical analysis

Baseline characteristics and number of medications prescribed were assessed separately for nursing home residents with and without dementia and compared using the chi-squared test and *t* test. We assessed the frequency and proportion of never appropriate medications for the 180–91-day period and the last 90 days prior to death. In another step, we assessed the frequency and proportion of newly prescribed and deprescribed medications. The chi-squared test was used to compare nursing home residents with and without dementia.

In addition, logistic regression was used to analyze the likelihood of new prescriptions of questionable medications within 90 days of death by age, sex, level of nursing care, number of prescribed medications, and length of stay in the nursing home. Odds ratios (OR) and 95% confidence intervals (95% CI) were calculated using a fully adjusted model with baseline characteristics and number of medications prescribed for all nursing home residents and stratified by dementia diagnosis.

All statistical analyses were performed using SAS for Windows version 9.4 (SAS Institute Inc., Cary, NC, USA).

## Results

### Baseline characteristics

A total of 29,052 (43.1%) of the 67,329 decedent nursing home residents were diagnosed with dementia. As shown in Table 1, residents with dementia tended to be older (average age at death of 86.4 years with dementia and 84.6 without dementia). Compared with residents without dementia, a higher proportion of nursing home residents with dementia had a nursing care level of 3. On average, nursing home residents without dementia were prescribed more medications than those with dementia (10.1 vs. 8.6). There was a higher proportion of women in both groups of nursing home residents.

**Table 1:** Baseline characteristics of nursing home residents (*n* = 67,328)

Characteristics	Dementia		*p* value^1^
	Present (%) (*n* = 29,052)	Not present (%) (*n* = 38,276)	
Age at time of death (mean age (SD))	86.4 ± 6.5	84.6 ± 7.9	< 0.0001
65–74 years	5.5	13.6	
75–84 years	28.7	29.5	
85–94 years	58.2	49.5	
≥ 95 years	7.6	7.3	< 0.0001
Sex			
Female	67.8	71.3	
Male	32.2	28.7	< 0.0001
Level of nursing care (at death)			
None/level 1	22.0	32.6	
Level 2	46.4	47.1	
Level 3	31.7	20.3	< 0.0001
Number of prescribed medications prior to death^2^ (mean number (SD))	8.6 ± 4.0	10.1 ± 4.8	< 0.0001
0–4	14.8	10.5	
5–9	47.0	38.2	
≥ 10	38.2	51.3	< 0.0001
Length of stay in nursing home			
25th percentile	75.0	33.0	
50th percentile	250.0	143.0	
75th percentile	598.0	488.0	< 0.0001

^1^Chi-squared test (categorical data) or *t* test (continuous data)

^2^Within 90 days of death

### Prescribed medication of questionable benefit

Table 2 shows the distribution of prescriptions of questionable medications in the 180–91-day period prior to death and the 90-day period prior to death between nursing home residents with and without dementia. Prescription of at least one medication of questionable benefit was more common among nursing home residents with dementia in the 180–91-day period prior to death (29.6% vs. 22.8%) as well as in the 90-day period prior to death (26.8% vs. 20.1%). When excluding anti-dementia medications, the prescription of medications of questionable benefit was less common among nursing home residents with dementia compared with those without dementia. Lipid-lowering agents were the most commonly prescribed medications of questionable benefit in both groups of nursing home residents. Acetylcholinesterase inhibitors and memantine were among the most commonly prescribed medications of questionable benefit for nursing home residents with dementia.

**Table 2:** Frequency (in percent) of inappropriate drug use in the 180 days and 90 days prior to death among nursing home residents, stratified by dementia status

Medications of questionable benefit	180–90 days prior to death		*p* value	90 days prior to death		*p* value
	With dementia	Without dementia		With dementia	Without dementia	
Acetylcholinesterase inhibitors	9.2	1.1	< 0.0001	7.6	1.1	< 0.0001
Memantine	6.1	0.8	< 0.0001	5.5	0.8	< 0.0001
Lipid-lowering agents	12.8	14.1	< 0.0001	11.5	12.1	< 0.05
Cytotoxic chemotherapy	0.0	0.4	< 0.0001	0.0	0.2	< 0.0001
Hormone antagonists	1.2	2.5	< 0.0001	1.1	2.1	< 0.0001
Antiplatelet agents^1^	4.3	4.6	< 0.05	4.4	4.7	0.056
Leukotriene inhibitors	0.1	0.1	0.103	0.0	0.1	< 0.01
Immunomodulators	0.4	1.1	< 0.0001	0.4	0.9	< 0.0001
Sex hormones	0.8	1.1	< 0.0001	0.6	0.8	< 0.001
≥ One medication of questionable benefit	29.6	22.8	< 0.0001	26.8	20.1	< 0.0001
≥ One medication of questionable benefit, excl. anti-dementia medication^2^	17.7	21.2	< 0.0001	16.4	18.5	< 0.0001

^1^Excluding aspirin

^2^Excluding acetylcholinesterase inhibitors and memantine

### Newly prescribed medications of questionable benefit

Table 3 shows the proportion of nursing home residents with newly prescribed medications of questionable benefit, stratified by dementia status. New prescription of at least one medication of questionable benefit prior to death was more common among nursing home residents with dementia compared with residents without dementia (9.8% vs. 8.7%). However, when excluding anti-dementia medication, new prescriptions of medication of questionable benefit were more common among nursing home residents without dementia (6.4% vs. 8.0%). Lipid-lowering agents were the most common newly prescribed medications of questionable benefit prior to death in both nursing home residents with dementia (4.4%) and without dementia (5.1%). Among nursing home residents with dementia, anti-dementia medications (acetylcholinesterase inhibitors (1.9%) and memantine (1.9%)) were the second most commonly newly prescribed medications.

**Table 3:** Frequency (in percent) of newly prescribed and deprescribed medications of questionable benefit prior to death among nursing home residents, stratified by dementia status

Medications of questionable benefit	Newly prescribed prior to death		*p* value	Deprescribed prior to death		*p* value
	With dementia	Without dementia		With dementia	Without dementia	
Acetylcholinesterase inhibitors	1.9	0.4	< 0.0001	3.5	0.5	< 0.0001
Memantine	1.9	0.3	< 0.0001	2.5	0.3	< 0.0001
Lipid-lowering agents	4.4	5.1	< 0.0001	5.7	7.1	< 0.0001
Cytotoxic chemotherapy	0.0	0.1	< 0.0001	0.0	0.2	< 0.0001
Hormone antagonists	0.4	0.7	< 0.0001	0.4	1.1	< 0.0001
Antiplatelet agents^1^	1.7	2.0	< 0.001	1.6	2.0	< 0.0001
Leukotriene inhibitors	0.0	0.1	< 0.01	0.0	0.0	0.235
Immunomodulators	0.1	0.4	< 0.0001	0.1	0.6	< 0.0001
Sex hormones	0.3	0.4	0.083	0.5	0.7	< 0.001
≥ One medication of questionable benefit	9.8	8.7	< 0.0001	13.2	11.6	< 0.0001
≥ One medication of questionable benefit, excl. anti-dementia medication^2^	6.4	8.0	< 0.0001	7.9	10.9	< 0.0001

^1^Excluding aspirin

^2^Excluding acetylcholinesterase inhibitors and memantine

Table 4 shows the association between different baseline characteristics and new prescriptions of medications of questionable benefit prior to death in the total study population and stratified by dementia status. Individuals with a dementia diagnosis were more likely to receive new medications of questionable benefit in the 90 days prior to death (OR 1.40, 95%CI 1.32–1.48). Excessive polypharmacy increased the risk for new prescriptions of medication of questionable benefit more than fourfold among all nursing home residents (OR 4.74, 95%CI 4.15–5.42).

**Table 4:** Adjusted odds ratios and 95% confidence intervals for new prescriptions of medication of questionable benefit by characteristics of nursing home residents

Characteristics	New prescription of medications of questionable benefit								
	With dementia			Without dementia			All		
	Frequency	(%)	OR (95% CI)	Frequency	(%)	OR (95% CI)	Frequency	(%)	OR (95% CI)
Age at death in groups									
65–74 years	207	13.0	REF.	476	9.1	REF.	683	10.0	REF.
75–84 years	1030	12.4	0.98 (0.83–1.15)	1147	10.1	1.28 (1.14–1.43)	2177	11.1	1.21 (1.11–1.33)
85–94 years	1510	8.9	0.74 (0.63–0.87)	1581	8.4	1.22 (1.10–1.37)	3091	8.6	1.04 (0.95–1.14)
≥ 95 years	106	4.8	0.44 (0.34–0.56)	123	4.4	0.75 (0.61–0.92)	229	4.6	0.64 (0.54–0.74)
Sex									
Female	1769	9.0	REF.	2196	8.1	REF.	3965	8.4	REF.
Male	1084	11.6	1.20 (1.10–1.30)	1131	10.3	1.30 (1.21–1.41)	2215	10.9	1.26 (1.19–1.33)
Level of nursing care									
None/level 1	775	12.2	REF.	1175	9.4	REF.	1950	10.3	REF.
Level 2	1368	10.2	0.84 (0.77–0.93)	1543	8.6	0.89 (0.82–0.96)	2911	9.2	0.87 (0.82–0.92)
Level 3	710	7.7	0.71 (0.64–0.79)	609	7.9	0.88 (0.80–0.97)	1319	7.8	0.79 (0.73–0.85)
Number of prescribed medications prior to death^1^									
0–4	156	3.6	REF.	86	2.1	REF.	242	2.9	REF.
5–9	1048	7.7	2.11 (1.78–2.51)	868	5.9	2.87 (2.30–3.59)	1916	6.8	2.39 (2.08–2.74)
≥ 10	1649	14.9	4.03 (3.40–4.77)	2373	12.1	5.94 (4.77–7.39)	4022	13.1	4.74 (4.15–5.42)
Time at nursing home quartiles									
100th (> 541 days)	541	6.6	REF.	551	6.4	REF.	1092	6.5	REF.
75th (190–540 days)	649	8.0	1.11 (0.99–1.25)	632	7.3	1.06 (0.94–1.20)	1281	7.6	1.09 (1.00–1.18)
50th (48–189 days)	903	12.0	1.55 (1.38–1.74)	789	8.6	1.15 (1.03–1.29)	1692	10.1	1.34 (1.23–1.45)
25th (< 47 days)	760	14.8	1.90 (1.68–2.14)	1355	11.5	1.57 (1.41–1.75)	2115	12.5	1.70 (1.57–1.84)
Dementia									
No							3327	8.7	REF.
Yes							2853	9.8	1.40 (1.32–1.48)

^1^Within 90 days prior to death

The effects of sex, level of nursing care, and number of medications prescribed on the risk of new prescription of medication of questionable benefit did not significantly differ between residents with and without dementia. However, nursing home residents with dementia aged 85–94 years and 95+ years were significantly less likely to receive prescriptions of questionable medication than nursing home residents without dementia (OR 0.74, 95%CI 0.63–0.87; OR 0.44, 95%CI 0.34–0.56). In contrast, nursing home residents without dementia aged 75 to 84 years and aged 85 to 94 years were at an excessive risk of receiving medications of questionable benefit (OR 1.28, 95%CI 1.14–1.43; OR 1.22, 95%CI 1.10–1.37).

### Deprescribing of medications of questionable benefit

In addition, Table 3 shows that a greater proportion of nursing home residents with dementia was subject to deprescription of medications of questionable benefit (13.2% vs. 11.6%). Lipid-lowering (5.7%) and also anti-dementia medications (acetylcholinesterase inhibitors, 3.5%; memantine, 2.5%) were the most commonly deprescribed medications of questionable benefit among nursing home residents with dementia.

## Discussion

### Main findings

Our longitudinal study among deceased nursing home residents showed that more than one quarter of the nursing home residents with dementia received medications of questionable benefit prior to death. When excluding anti-dementia medications, a lower proportion of nursing home residents with dementia received medications of questionable benefit than nursing home residents without dementia. However, more than 16% of nursing home residents with a dementia diagnosis still received inappropriate medications, and 6.4% even received new prescriptions, i.e., new treatment initiations in the 90 days prior to death. Of these new prescriptions, lipid-lowering agents were the most commonly prescribed. Residents with dementia were at increased risk for new prescriptions of medications of questionable benefit, even when age, level of nursing care, number of medications, and time since nursing home admission were considered.

### Comparison with other studies

Our study used the Holmes et al. criteria employed by previous studies on questionable medication prescriptions in dementia [13–15, 17, 24]. Our results on residents with dementia are comparable with those of a Swedish study by Morin et al., which reported that 32.8% of nursing home residents with dementia received medications of questionable benefit prior to death [14]. The study population by Morin et al. had a higher proportion of residents aged 85+ years (72.4%) than our population (65.8%). In addition, our study included those aged 65 to 74 years and considered exclusively nursing home residents. Another study conducted by Matlow et al. in a Canadian setting reported up to 86.3% of nursing home residents with dementia receiving at least one medication of questionable benefit in the 120-day period prior to death [15]. However, this study included only nursing home residents already subject to at least one medication of questionable benefit in the last year of life. Similar to Morin et al. and Matlow et al., we found a decline in the prevalence of medications of questionable benefit among nursing home residents closer to death [14, 15].

The most commonly prescribed questionable medications came from the group of lipid-lowering agents. Yet, the benefit of lipid-lowering agents in patients with dementia is questionable. Studies have shown that these medications reduce the quality of life in terminally ill patients and are safe to discontinue [25, 26]. New initiation of these medications, shown to take place in our study, should therefore be carefully considered. Lipid-lowering agents are associated with a risk of adverse effects, and their benefits accrue only after a prolonged time [25]. Thus, the benefits of lipid-lowering agents are limited and may not outweigh the impact on quality of life among older people in palliative care settings [27, 28]. Further research and direct guidelines may be needed to regulate new lipid-lowering agent prescriptions among nursing home residents with advanced dementia [29].

Anti-dementia drugs, particularly acetylcholinesterase inhibitors, were among the most commonly prescribed medications of questionable benefit, a finding which is in line with previous studies [14–16]. Acetylcholinesterase inhibitors are indicated for mild to moderate dementia in order to attenuate the manifestation of dementia [30–33]. However, the indication for acetylcholinesterase inhibitors among nursing home residents with advanced dementia is less clear, and their use in this population is considered off label in Germany [33–36]. Despite the questionable benefits, discontinuation of acetylcholinesterase inhibitors may be difficult and requires individualized approaches [13, 37]. However, our study also showed that 1.9% of nursing home residents with dementia received a new acetylcholinesterase inhibitor, i.e., initiation of this medication in the last 90 days prior to death. New prescription of this medication requires careful consideration given the potential adverse effects [38], decrease in quality of life [39], and primary treatment indication for mild to moderate dementia [32]. For residents with moderate to severe dementia, memantine is an approved option that can be combined with acetylcholinesterase inhibitors [40]. However, the clinical benefits of memantine in advanced dementia are limited, and its side-effect profile needs to be carefully considered in end-of-life care [41–43]. In addition to the prevalent prescription among residents with dementia, anti-dementia medications were also prescribed among residents without dementia. This may be related to the treatment of dementia secondary to other organic causes or isolated treatment of dementia-related symptoms like memory loss among residents without a formal dementia diagnosis [44].

Nursing home residents with a dementia diagnosis were more likely to receive at least one new prescription of questionable medications prior to death compared with residents without dementia, which is mainly attributable to the prescription of anti-dementia medications. In addition, polypharmacy, i.e., the concurrent use of 5 or more medications, was strongly associated with new prescriptions of questionable medication, while residents of older age and a higher level of nursing care were less likely to receive such new prescriptions. These results mirror previous findings and may reflect an earlier and more targeted initiation of palliative approaches for older and more morbid nursing home residents [15]. Residents with dementia and polypharmacy, those of younger age, i.e., 65 to 74 years, and those with a low nursing care level were more likely to be prescribed new medications of questionable benefit. New prescriptions may be related to a lack of guidelines, misjudgment of disease progression, and difficulties estimating life expectancy among these residents with advanced dementia [2, 45, 46]. This may lead to varying treatment approaches, including the initiation of new anti-dementia medications at the end of life [24, 47, 48]. Studies have pointed to the use of prognostic models when estimating life expectancy among individuals with dementia [45, 46, 49]. Assessing the life expectancy of nursing home residents with advanced dementia can also guide the clinical decision-making process in prescription regimens. The use of prognostic tools, alongside advance care planning and in consideration of the nursing home resident’s preferences, can then assist palliative care decisions. These decisions may involve a greater focus on improved comfort and quality of life, including the minimization of interventions like tube feeding or medication prescriptions [16, 50–52].

In residents both with and without dementia, men were more likely to receive new medications of questionable benefit prior to death. A previous study has pointed toward sex-specific differences in dementia care, with men more commonly receiving burdensome interventions at the end of life [53]. The differences between men and women may be more related to social contexts than biological phenomena, i.e., gender-based differences in the provision of care or different attitudes toward death [53–55].

### Strengths and limitations

One of the major strengths of this study is its sample size. We were able to assess 67,328 deceased nursing home residents and their respective medication claims prior to death. In addition, information obtained from claims data has been shown to be of high accuracy [56]. To the best of our knowledge, no previous study reported similar findings in the context of Germany or considered new prescriptions of these medications prior to death.

However, there are also limitations to our study that need to be considered. Information on dementia diagnoses was limited to the details available in the claims data. Misclassification of some residents with dementia is possible, with residents receiving no dementia diagnosis but anti-dementia medication. In addition, unreported or undiagnosed individuals, particularly those with mild presentations, may not have been included, leading to an underestimation of dementia cases and the use of questionable medications in this group. The selected sample may primarily reflect nursing home residents with typical signs and thus advanced levels of dementia.

The utilized list of “never appropriate” medications from Holmes et al. focuses on individuals with advanced dementia [13]. We used this list in our assessment of all nursing home residents, comparing residents with and without dementia prior to death. However, we employed this list with and without anti-dementia drugs.

The assessed data stem from a single health insurance provider in Germany. It needs to be considered that German insurance funds differ in regard to sex, age, socioeconomic backgrounds, and morbidity. DAK-Gesundheit covers a relatively high proportion of women as well as individuals with higher levels of morbidity [57]. These differences need to be considered regarding the generalizability of data to the total German population.

## Conclusion

This study assessed the prevalence of medications of questionable benefit among nursing home residents with and without dementia prior to death, showing that more than one-fourth of nursing home residents with dementia and limited life expectancy received these medications compared with one-fifth of residents without dementia. The number of nursing home residents with advanced dementia and limited life expectancy receiving these medications is considerable, even when accounting for anti-dementia medication. Physicians may consider nursing home residents’ life expectancy when prescribing new medications, particularly those of questionable benefit.

## Electronic supplementary material

ESM 1 (DOCX 14 kb).
