# Supplementary material for: Prescribing medications of questionable benefit prior to death: a retrospective study on older nursing home residents with and without dementia in Germany
Source: Eur J Clin Pharmacol. 2020 Mar 26;76(6):877–85. doi: 10.1007/s00228-020-02859-3 (PMC7239800; doi:10.1007/s00228-020-02859-3)
Supplement: Supplementary file 1 — (DOCX 14 kb). [file 228_2020_2859_MOESM1_ESM.docx]

| **Suppl. Table 1.** Medications considered to be never appropriate for prescribing in older people with advanced dementia, according to Holmes et al. | |
| --- | --- |
| **Medication name** | **ATC** |
| acetylcholinesterase inhibitors | N06DA |
| Memantine | N06DX01 |
| lipid-lowering agents | C01A |
| cytotoxic chemotherapy | L01D |
| hormone antagonists | L02B |
| antiplatelet agents1 | B01AC |
| leukotriene inhibitors | R03DC |
| immunomodulators | L03-L04 |
| sex hormones | G03 |

Supplementary Table 1. Medications considered to be never appropriate for prescribing in older people with advanced dementia, according to Holmes et al.
